# Metabolic Modulation in Macrophage Effector Function

**DOI:** 10.3389/fimmu.2018.00270

**Published:** 2018-02-19

**Authors:** Ciana Diskin, Eva M. Pålsson-McDermott

**Affiliations:** ^1^School of Biochemistry and Immunology, Trinity College Dublin, Trinity Biomedical Science Institute, Dublin, Ireland

**Keywords:** macrophage, immunometabolism, glycolysis, tricarboxylic acid cycle, electron transport chain

## Abstract

Traditionally cellular respiration or metabolism has been viewed as catabolic and anabolic pathways generating energy and biosynthetic precursors required for growth and general cellular maintenance. However, growing literature provides evidence of a much broader role for metabolic reactions and processes in controlling immunological effector functions. Much of this research into immunometabolism has focused on macrophages, cells that are central in pro- as well as anti-inflammatory responses—responses that in turn are a direct result of metabolic reprogramming. As we learn more about the precise role of metabolic pathways and pathway intermediates in immune function, a novel opportunity to target immunometabolism therapeutically has emerged. Here, we review the current understanding of the regulation of macrophage function through metabolic remodeling.

## Introduction

All living cells rely on an organized sequence of anabolic and catabolic reactions to produce a steady supply of energy and biosynthetic precursors. In order to optimize functionality, enzymes that control these tightly regulated metabolic pathways are compartmentalized into specific organelles within the cells. Immune cells such as macrophages are no different in this aspect; however, recent studies now reveal that immune effector functions such as cytokine production in response to pathogens are directly coupled to specific changes in cellular metabolism. This metabolic reprogramming of immune cells is required for both inflammatory and anti-inflammatory responses.

Macrophages are found in almost every tissue in our body, and along with dendritic cells they are at the forefront of initiating an innate immune response through phagocytosis and cytokine release, as well as an adaptive immune response through antigen presentation. Recognized nomenclature divides activated macrophages into two subgroups *in vitro*: the classically activated macrophages (M1) associated with inflammatory responses, which *in vitro* are generated by typically stimulating the resting macrophages with lipopolysaccharide (LPS) and interferon gamma (IFN-γ). Secondly, the alternatively activated macrophages (M2) are associated with tissue remodeling, resolution of inflammation, and anti-inflammatory responses, and are generated *in vitro* using anti-inflammatory stimuli including IL-4. We now know that this is an oversimplification of the actual functional diversity occurring *in vivo*. The vast spectrum of different macrophage activation statuses was clearly demonstrated in a transcriptomics study by Xue et al. who stimulated human macrophages with a range of stimuli (1). In addition, gene-set enrichment analysis was applied to sample groups from smokers and COPD patients. The data set generated, and other transciptome studies published since, proposes a spectrum model of macrophage activation rather than the dichotomous M1/M2 classification system. While useful in mapping the metabolic pathways of differentially activated macrophages, and although many of the studies described here classify macrophages as M1 or M2, we now view macrophage polarization differently. Evidenced primarily *in vivo*, macrophages respond to specific external stimuli, resulting in unique sets of macrophage phenotypes that fall between the two extremes of M1 and M2. Hence, manipulating or skewing the different macrophage phenotypes in clinical settings such as asthma, sepsis, tumor, atherosclerosis, infectious disease, and metabolic disorders may provide us with a novel therapeutic approach.

Here, we review current literature on how macrophages utilize metabolic pathways in order to generate adequate energy and biosynthetic macromolecules to meet the fluctuating needs involved in host immune responses.

## Glycolysis

### Overview

**Figure 1:**
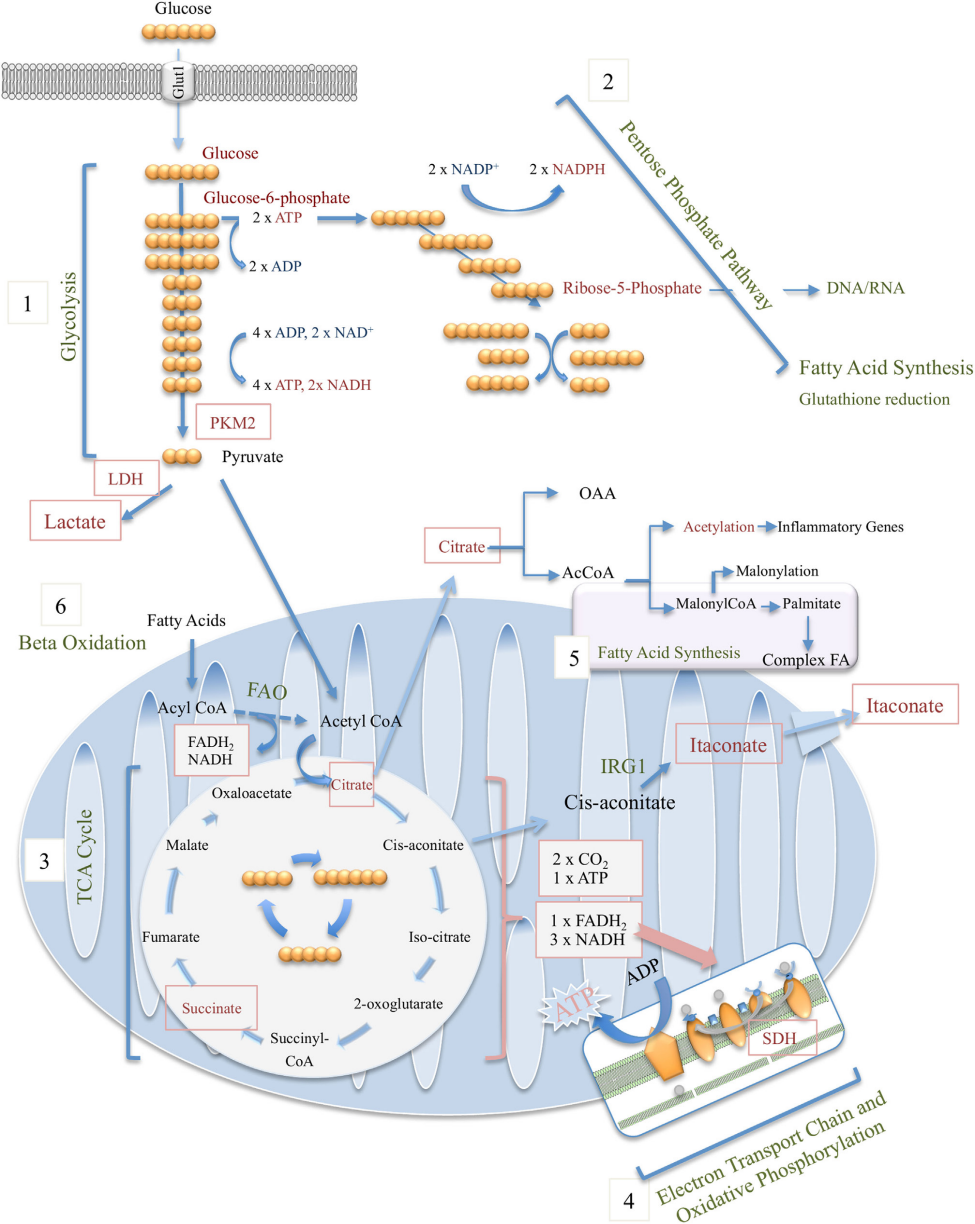
Overview of macrophage metabolic pathways, including glycolysis (1), pentose phosphate pathway (2), tricarboxylic acid (TCA) cycle (3), electron transport chain and oxidative phosphorylation (4), fatty-acid synthesis (5), and beta (fatty acid) oxidation (6).

Glucose, fructose, pyruvate, and other small carbohydrates play key roles in energy metabolism as well as provide carbon skeletons for the synthesis of other macromolecules. Glycolysis, the process which involves the breakdown of six-carbon glucose to three-carbon pyruvate, is central in generating ATP without requiring oxygen, where the reverse process, gluconeogenesis, consumes ATP while generating polysaccharides for storage. Glycolysis involves 10 enzymatically regulated steps, overall generating two molecules of pyruvate per molecule of glucose, with a net energy gain of two ATP and two NADH (Figure 1). Although often illustrated as a linear reaction, in fact glycolysis branches off in order for intermediate metabolites to proceed along other metabolic pathways. These include the first intermediate of glycolysis, glucose-6-phosphate, which is required for glycogen synthesis and the pentose phosphate pathway (PPP), as well as the glycolytic intermediate glyceraldehyde-3-phosphate, which through glycerol generates triglycerides and fatty acids (Figure 1).

For many years, the general school of thought has been that pyruvate generated through glycolysis enters the mitochondria where it undergoes oxidative decarboxylation by pyruvate dehydrogenase (PDH) and thereby serves as the major source of acetyl-CoA, the starting point of the tricarboxylic acid (TCA) cycle (see below). During periods of high energy demand or low oxygen supply, pyruvate can instead be converted to lactate, another potential nutrient, by lactate dehydrogenase (LDH). However, new data by Hui et al. beg us to rethink the role for lactate in fueling the TCA cycle (2). Instead of pyruvate feeding the TCA cycle, circulating lactate was instead shown to be the primary source of carbon for the TCA cycle in all tissues except for brain.

### Glycolysis and Macrophage Function

The upregulation of glycolysis in activated macrophages was first observed several decades ago (3–5), but it is only in recent years that the mechanisms governing the process and the functional significance of this metabolic shift have been unearthed. A sharp increase in the rate of glycolysis is now closely associated with an inflammatory phenotype in macrophages. It has been demonstrated that administration of 2-deoxyglucose (2-DG), a derivative of glucose that is taken up by the cell but cannot be further processed, blocks many aspects of the classical M1 inflammatory phenotype including opsonin-mediated phagocytosis (6, 7), secretion of pro-inflammatory cytokines, and production of reactive oxygen species (ROS) (8). In contrast, alternatively activated M2 macrophages or those associated with immune tolerance, such as alveolar macrophages, employ oxidative phosphorylation (OXPHOS) over glycolysis as their main source of ATP (5). The rapid increase in glucose uptake by classically activated M1 macrophages is facilitated by upregulation of glucose transporter 1 (GLUT1) expression (8, 9) (Figure 1).

It may seem counterintuitive that activated macrophages utilize glycolysis as their main source of energy, as OXPHOS generates 36 molecules of ATP compared with glycolysis, which produces a mere two molecules of ATP per molecule of glucose. However, a substantial boost in glycolysis can be achieved more rapidly than in OXPHOS, which would require concomitant mitochondrial biogenesis. Not only does glycolysis confer an advantage in terms of the speed at which it can be upregulated, but also it provides biosynthetic intermediates to be used in the PPP, among other processes, which are hugely important for classical macrophage activation and effector functions [reviewed in Ref. (10, 11)].

Multiple studies using murine and human macrophages have demonstrated that classically activated M1 macrophages are heavily dependent on glycolysis. Rodriguez-Prados et al. used a glucose tracer-based metabolomics approach to show that activation of murine peritoneal macrophages through various toll-like receptor (TLR) pathways all resulted in a highly glycolytic phenotype (12). It was also determined using extracellular flux analysis to study metabolic features of murine M1 and M2 macrophages, that M2 macrophages display enhanced mitochondrial OXPHOS, whereas M1 macrophages predominantly use glycolysis to generate ATP (13). Human studies seem to mimic observations in mice, with the leukocytes of patients suffering from sepsis undergoing a shift to aerobic glycolysis, which is reversed upon patient recovery (14). In contrast to LPS and other pro-inflammatory stimuli enhancing aerobic glycolysis in macrophages, anti-inflammatory signals have been shown to exert the opposite effect on macrophage glucose metabolism. It was recently established that interleukin (IL)-10 suppresses glycolysis in LPS-stimulated wild-type bone-marrow-derived macrophages (BMDMs). Furthermore, in contrast BMDMs derived from *Il10^−/−^* mice exhibit elevated rates of glycolysis (15). The effect of IL-10 on glycolysis may be dependent on nitric oxide (NO) (16).

The elevated glycolysis associated with inflammatory macrophages is heavily dependent on hypoxia-inducible factor-1 (HIF-1α). When oxygen levels are low, HIF-1α no longer undergoes prolyl hydroxylation, leading to a decreased binding of the interacting partner von Hippel–Lindau protein (VHL), and reduced proteosomal degradation of HIF-1α. As a result, stabilized HIF-1α can bind the constitutively expressed HIF-1β subunit, initiating the transcription of hypoxic genes, including glucose transporters and glycolytic enzymes (17–23). Blouin et al. were first to show that stimulation of macrophages with LPS increased HIF-1α protein levels, leading to a functional HIF-1 complex that bind to hypoxic response elements (HREs) in target genes (24). It was later determined that the induction of HIF-1α in the context of inflammation was dependent on NF-κB, which acts as a transcriptional activator of HIF-1α (25). HIF-1α was also found to play a role in trained immunity, which involves epigenetic remodeling of myeloid cells in response to stimuli such as β-glucan (26). β*-*Glucan derived from *Candida Albincans* plays a central role in the induction of innate immune memory and is known to confer protection against a range of infections. It was also found that the HIF-1α glycolytic reprogramming of activated macrophages played a significant role in monocyte-derived macrophage migration into tissues (27). HIF-1α also induces the transcription of the key pro-inflammatory cytokine IL-1β (28).

Although a Warburg-like phenomenon is predominantly associated with M1 macrophages, alternatively activated macrophages also display an upregulated rate of glycolysis in addition to augmented mitochondrial metabolism. Huang et al. found that both IL-4 and macrophage colony-stimulating factor (M-CSF) drive mechanistic target of rapamycin complex 2 (mTORC2) activation, which in turn induces interferon regulatory factor 4 (IRF4) expression, contributing to the upregulation of glycolysis (29). Another study found that M-CSF, which is associated with M2 polarization, instigated a similar expression of glucose transporters, a higher lactate production rate, and increased expression of several glycolytic enzymes in macrophages, compared with granulocyte-macrophage colony-stimulating factor (GM-CSF), which is typically associated with M1-like phenotype (30). Tan et al. found that administration of 2-DG in addition to IL-4 reduced the expression of early M2 activation markers (31). As more studies like these emerge, it may transpire that glycolysis could play a more important role in M2 macrophages than previously considered.

### Multiple Roles of Glycolytic Enzymes

Specific roles for several of the glycolytic enzymes have been identified in macrophages. Some of these enzymes “moonlight” by carrying out functions in immunity separate to their glycolytic activity. One example includes hexokinase, which has been found to not only function as a pattern recognition receptor (PRR), but also, together with mTORC1, plays a critical role for nod-like receptor family pyrin domain containing 3 (NLRP3) inflammasome assembly (32). Furthermore, a component of bacterial peptidoglycan, *N*-acetylglucosamine, can bind to hexokinase, resulting in its inhibition and subsequent dissociation from the outer membrane of the mitochondria, culminating in NLRP3 activation (33).

Glyceraldehyde-3-phosphate dehydrogenase (GAPDH) can bind to AU-rich RNA sequences in its Rossman fold, the site that typically binds NAD^+^ (34). GAPDH takes part in formation of the gamma–interferon-activated inhibitor of translation (GAIT) complex. Upon assembly in murine macrophages, the GAIT complex binds to a specific element in the 3′untranslated region (3′UTR) of several inflammatory mRNAs and inhibits their translation. Its targets include vascular endothelial growth factor (VEGF), several chemokines, and corresponding chemokine receptors (35). GAPDH alone can bind directly to mRNA to inhibit translation of IFN-γ in T cells *via* 3′UTR binding (36) and also block translation of tumor necrosis factor-α (TNF-α) mRNA in human macrophages (37).

α-enolase is another glycolytic enzyme that was found to display non-glycolytic functions in macrophages. Bae et al. found that monocytes and macrophages in the inflamed synovium of rheumatoid arthritis (RA) patients and in a mouse model of arthritis expressed surface α-enolase. Antibodies against enolase, previously reported in RA patients, were shown to increase the production of pro-inflammatory cytokines and prostaglandins from enolase-expressing macrophages and therefore may contribute to the pathogenesis of the disease (38).

Another glycolytic regulator that is of great importance in macrophage effector functions is pyruvate kinase M2 (PKM2). PKM2 is an HIF-1α target gene that was originally found to promote the Warburg effect in tumor cells. It was also found to interact directly with HIF-1α in the nucleus and enhance the transcription of HIF-1α-responsive genes (39, 40). PKM2 was later found to play a significant role in LPS-activated macrophages. Dimeric enzymatically inactive PKM2 translocates to the nucleus where it acts as a coactivator of HIF-1α, promoting expression of pro-inflammatory as well as pro-glycolytic genes. Nuclear PKM2 together with HIF-1α binds directly to HRE sites in the IL-1β promoter in LPS-stimulated macrophages (41). Using small-molecule activators that promote a tetrameric form of PKM2, the pyruvate kinase enzymatic activity can be restored while simultaneously preventing nuclear translocation. PKM2 activators impaired M1 macrophage polarization, promoting expression of M2 genes while reducing LPS-induced glycolysis. Furthermore, these activators diminished IL-1β production *in vivo* in response to *Salmonella typhimurium* or LPS alone and increased levels of anti-inflammatory IL-10. PKM2 was also found to play a role in NLRP3 and absent in melanoma 2 (AIM2) inflammasome activation. It was demonstrated that PKM2-dependent glycolysis promotes the phosphorylation of eukaryotic translation initiation factor-2 alpha kinase 2 (EIF2AK2, also called PKR), which was previously shown to be necessary for inflammasome activation and secretion of IL-1β, IL-18, and high-mobility group box 1 protein (HMGB1) from macrophages (42, 43). PKM2 may also contribute to the pathogenesis of coronary artery disease. Peripheral circulating monocytes differentiated *ex vivo*, as well as macrophages from the atherosclerotic plaques of patients suffering from coronary artery disease exhibit increased expression of dimeric PKM2, augmented glycolytic flux, and upregulated ROS production. PKM2 translocates to the nucleus and phosphorylates signal transducer and activator of transcription 3 (STAT3), contributing to the increase in IL-1β and IL-6 associated with these patients (44). Very recently, PKM2 was shown to regulate the expression of the checkpoint programmed death-ligand 1 (PD-L1), a ligand for the immune checkpoint receptor PD-1, in macrophages as well as other immune cells and cancer cells. Both pharmacological intervention and genetic silencing of PKM2 inhibited LPS-induced expression of PD-L1. Furthermore, PKM2 and HIF-1α bind to two HRE sites in the promoter of PD-L1 (45). This observation could have therapeutic potential as targeting immune checkpoints such as PD-L1 and PD1 has proven successful clinically [reviewed in Ref. (46)].

### Tumor-Associated Macrophages (TAMs)

In cells undergoing Warburg metabolism, pyruvate resulting from glycolysis is diverted away from the TCA cycle and instead becomes converted to lactate by LDH. In addition to macrophages producing lactate, extracellular lactate from surrounding tissues also impact on macrophage function. Lactate secreted from tumor cells was found to drive M2 polarization in TAMs, which facilitated tumor growth (47). Although TAMs are often considered to be more M2-like, we now know that they have a high glycolytic rate similar to M1 macrophages; however, the effect of this on tumor progression is somewhat unclear (48). Murine TAMs exhibit diminished glycolysis through expression of REDD1, an mTORC1 inhibitor. This decrease in glycolysis is thought to facilitate metastasis and aberrant angiogenesis in tumors (49). However, a study carried out using TAMs generated *in vitro* from human monocytes yielded quite different results. They found an elevated glycolytic flux in TAMs to be associated with angiogenesis and metastasis in pancreatic cancer and showed that treatment with 2-DG was sufficient to reverse this effect (50). TAM metabolism is undoubtedly complicated and this area of research was extensively reviewed recently (51).

## The Pentose Phosphate Pathway (PPP)

### Overview

Glucose-6-phosphate from glycolysis feeds the anabolic PPP, which not only generates pentoses and 5-ribose phosphate for nucleic acid production but also serves as our major source of NADPH (Figure 1). NADPH provides the reducing power required for a range of synthetic reactions and anabolic pathways. NADPH offers reducing equivalents for generation of the antioxidant glutathione, thereby allowing for clearance of harmful ROS as well as being responsible for the respiratory burst in neutrophils and macrophages generating H_2_O_2_ to aid bacterial killing.

Like glycolysis, the PPP takes place in the cytosol and can be divided into an initial oxidative phase during which NADPH is generated, and a later non-oxidative phase where five-carbon sugars are synthesized.

### PPP and Macrophage Function

The PPP has been shown to be upregulated in M1 macrophages (28, 52). NAPDH is likely to be of great importance for M1 macrophages as it is required by the enzyme NADPH oxidase which catalyzes the generation of ROS. As mentioned, NADPH is also used for the production of antioxidants, which may be important in the resolving phase of inflammation (53). Production of nucleotides is likely to be essential for activated macrophages. Although they display a reduced rate of proliferation, nucleotides are required for miRNAs involved in gene regulation (11). M2 macrophages, on the other hand, appear to suppress the PPP. Haschemi et al. demonstrated that regulation of the PPP in macrophages is under the control of the carbohydrate kinase-like protein (CARKL), a sedoheptulose kinase. CARKL was found to be upregulated in response to IL-4 but suppressed in response to LPS, resulting in an inhibition of the PPP in M2 macrophages (54). This conclusion was drawn from experiments using primary murine macrophages, human peripheral blood mononuclear cells (PBMC), and the macrophage cell line RAW 264.7. Employing overexpression and genetic silencing in RAW 264.7 cells, the authors found that the loss of CARKL mimicked the increase in extracellular acidification rate (ECAR) and decrease in oxygen consumption rate (OCR) that is seen upon LPS stimulation, while overexpression attenuated the effect that LPS has on the ECAR and OCAR. Therefore, the downregulation of CARKL seems to be important for the redirection of glucose from aerobic metabolism to glycolysis and the PPP that is seen in pro-inflammatory macrophages.

## The Tricarboxylic Acid (TCA) Cycle

### Overview

When oxygen is readily available, glycolysis becomes the initial stage of glucose catabolism. Once pyruvate and lactate are generated, three further metabolic processes occurring in the mitochondria become responsible for potentially generating a further 36 molecules of ATP per glucose molecule. Firstly, pyruvate is oxidized through a series of reactions termed the TCA cycle. This is followed by the electron transport chain (ETC), and lastly the OXPHOS of ADP to ATP, a process that is driven by the proton gradient resulting from electron transport (Figure 1).

The point of entry for pyruvate formed during glycolysis into the TCA cycle comes when pyruvate is decarboxylated to acetyl CoA by the PDH complex. Acetyl CoA then enters a series of eight enzymatically regulated oxidizing reactions where each acetyl CoA is converted into two molecules of water and carbon dioxide. Pyruvate loses one-carbon and the two-carbon acetyl group of acetyl CoA condenses with the acceptor compound oxaloacetate resulting in six-carbon citrate. In a cyclic, carefully regulated series of reactions citrate is decarboxylated and oxidized resulting in malate from which the starting oxaloacetate is regenerated, completing the cycle (Figure 1). Only one single ATP is directly generated by one lap around the TCA cycle (two per molecule of glucose); however, most of the energy produced is stored in the form of the reduced coenzymes NADH and FADH_2_ which can drive the production of large amounts of ATP in the subsequent reactions of the ETC and OXPHOS (see below). In contrast to glycolysis the TCA cycle requires oxygen.

### TCA Cycle and Macrophage Function

In addition to the increased glycolytic flux and reduced oxygen consumption that have been extensively studied in inflammatory macrophages for several decades (55), significant changes are also known to occur in the TCA or Krebs cycle. As with glycolysis, key intermediates of the TCA cycle serve as precursors in biosynthetic pathways. Citrate plays an important role here, fueling not only fatty-acid synthesis and histone acetylation but also acts as a precursor of itaconate, one of the most highly induced metabolites in LPS-activated macrophages. Citrate is firstly converted into *cis*-aconitate by mitochondrial aconitase 2 (ACO2), which is turned into itaconate by immune-responsive gene 1 (IRG1), also known as *cis*-aconitate decarboxylase (CAD) (Figure 1).

Resting macrophages and M2-like macrophages are considered to utilize an intact TCA cycle in conjunction with OXPHOS in order to generate ATP. An intact TCA cycle is thought to be important for the UDP-GlcNAc-mediated glycosylation of lectin and mannose receptors that are highly expressed on M2 macrophages (52). Pyruvate generated in M1 macrophages is converted to acetyl CoA by PDH, which is later converted to citrate. PDH activity was found to be intact in M1 macrophages, even though HIF-1α can potentially induce pyruvate dehydrogenase kinase 1 (PDK-1), an inhibitor of PDH (56). Interestingly, as mentioned, recent data have established that glucose fuels the TCA cycle indirectly *via* circulating lactate. Using ^13^C-labeled lactate and other metabolites, the authors show that the carbons in TCA cycle intermediates in most tissues arose from circulating lactate, instead of directly from glycolysis (2). As inflammatory macrophages are known to produce large quantities of lactate, it is plausible that this lactate could be important for use in the TCA cycle in surrounding tissues or even other immune cells. However, it remains to be confirmed if these new data have any implications for macrophage metabolism.

When macrophages are stimulated with LPS or another inflammatory signal, their TCA cycle becomes disrupted at distinct points in the cycle (52, 57). Therefore, an accumulation of certain metabolites such as citrate, itaconate, and succinate occurs in M1 macrophages.

### Citrate

M1 macrophages display increased levels of citrate giving a first indication to its importance in macrophage effector functions. This accumulation in citrate is likely due to a downregulation of isocitrate dehydrogenase (IDH), the enzyme that catalyzes the conversion of isocitrate to α-ketoglutarate (52). An early study also reported enhanced activity of citrate synthase, the enzyme that catalyzes the formation of citrate from acetyl CoA and oxaloacetate (3). Both mRNA levels and protein expression of the mitochondrial citrate carrier (CIC, also termed Slc25a1) were found to be elevated in LPS-stimulated macrophages. CIC exports citrate from the mitochondrial matrix while importing cytosolic malate (58). In addition, the same group later demonstrated that CIC is upregulated in response to the pro-inflammatory cytokines TNF-α and IFN-γ (59). Once in the cytosol, citrate can be used for *de novo* lipogenesis (discussed below), which is important for membrane biogenesis. This involves the conversion of citrate to acetyl CoA by ATP citrate lyase (ACLY) (60). Interestingly, ACLY activity was found to be regulated by IL-4 *via* Akt-mTORC1 signaling. This alters histone acetylation and therefore regulates the expression of a subset of M2-associated genes (61). Citrate also appears to be critical for prostaglandin production, as both pharmacological inhibition and knockdown of CIC markedly reduced prostaglandin E2 (PGE2) levels (59). Disrupting ACLY activity also led to a significant decrease in PGE2 (60). In addition to acting as a pro-inflammatory mediator itself, PGE2 was also recently shown to be essential for LPS-induced expression of pro-IL-1β (62). Perturbing the activity and/or expression of CIC or ACLY also negatively affects NO and ROS production (58–60). Genetic silencing of ACLY and the use of three different inhibitors all reduce levels of ROS and NO in the human cell line U937. Similarly, knockdown or chemical inhibition of CIC also significantly reduced levels of ROS and NO. Although the mechanism is not yet fully elucidated, authors propose that ACLY activity may play a role in ROS and NO production through indirectly boosting NADPH supplies.

Citrate accumulation in macrophages can also lead to changes in gene expression. Citrate-derived acetyl CoA is critical for histone acetylation, as this process was found to be impaired upon siRNA-induced silencing of ACLY. These epigenetic changes could be of great importance in the context of inflammation; for example, IL-6 expression has been shown to be regulated by histone acetylation in macrophages (63). Non-histone protein acetylation can also impact cytokine expression, as microtubule acetylation was found to modulate IL-10 production (64). Citrate has also been identified as an inhibitor of HIF asparaginyl hydroxylase (FIH), which acts as a negative regulator of HIF-1α activity. Hence, citrate could potentially indirectly regulate HIF-1α targeted genes (65). However, none of the studies linking citrate to acetylation of proteins have yet been carried out in macrophages.

Citrate-derived acetyl CoA can also be converted to malonyl CoA, which acts as a cofactor for a lysine modification dubbed malonylation. This modification changes a positively charged residue into a negative charge (66, 67). This modification, although only recently discovered, has already been implicated in type-2 diabetes (68). Malonylation has not yet been documented in macrophages or other immune cells but could potentially play a role, given the accumulation of citrate observed in M1 macrophages.

### Itaconate

Citrate-derived *cis*-aconitate can be converted to itaconate, one of the most highly induced metabolites in activated macrophages (52, 56, 69). Although itaconate has been gaining more interest in recent years, its antibacterial effects have been recognized since the 1970s when it was shown to inhibit the growth of *Pseudomonas indigofera* by targeting isocitrate lyase––an important enzyme in the glyoxylate cycle in bacteria (70). More recently, itaconate has been shown to exert bacteriostatic effects on *Mycobacterium tuberculosis, S. enterica* (71), and *Legionella pneumophila* (72). There is also evidence of bacteria evolving to combat the action of this immunometabolite, as *P. aeruginosa* and *Yersinia pestis* were both found to express three separate enzymes that function in the degradation of itaconate. These three genes were found to be critical for the pathogenicity and survival of these bacteria (73). Itaconate was also implicated in a pro-inflammatory setting in two metabolic screens: one carried out in mice infected with *M. tuberculosis* (74) and the other in the macrophage-like cell line RAW 264.7 stimulated with LPS (75).

Immune-responsive gene 1, also later known as CAD/ACOD1, was previously known to be induced upon stimulation with LPS (76), but its function was not elucidated until Michelucci et al. demonstrated that it was the enzyme responsible for catalyzing the decarboxylation of *cis*-aconitate to produce itaconate. As expected, knocking down IRG1 in a macrophage cell line resulted in impaired antibacterial activity, due to a significant drop in itaconate levels (71). In addition to studies showing an increase in IRG1 expression in murine M1 macrophages, it has also been shown to be upregulated in humans during sepsis (77). Although the bactericidal effects have been well characterized, the immunomodulatory function of itaconate is a more recent area of study. Itaconate is generally thought of as being anti-inflammatory and was shown to inhibit the production of several TLR-induced pro-inflammatory cytokines by augmenting the expression of A20, a negative regulator of NF-κB (77). IRG1 was also found to be induced by the activity of heme oxygenase 1 (HO-1), which is expressed in the lungs and associated with LPS tolerance. Induction of HO-1 with carbon monoxide was found to decrease TNF-α levels and inhibition of HO-1 had the opposite effect, providing further evidence for the anti-inflammatory effects of IRG1 (78). IRG1 has also been shown to play a role in implantation, a process generally associated with immune tolerance or immune suppression. The expression of IRG1 was demonstrated to be regulated by the progesterone receptor (79) and leukemia inhibitory factor (LIF) (80), both of which are heavily involved in the implantation process.

Itaconate was found to associate with the mitochondria (81) and was shown to reduce mitochondrial substrate-level phosphorylation, an effect that was abrogated upon siRNA-mediated silencing of IRG1 (82). Itaconate has been proposed to contribute to the second breakpoint in the TCA cycle that occurs in M1 macrophages. Through its ability to inhibit succinate dehydrogenase (SDH), the enzyme that catalyzes the oxidation of succinate to fumarate, overproduction of itaconate leads to an accumulation of succinate (83, 84). This increase in succinate levels was abolished in *Irg1^−/−^* macrophages, whereas treatment with exogenous itaconate in the form of dimethyl itaconate (DMI) was found to enhance succinate levels (83). Furthermore, by using exogenous itaconate, as well as using mice lacking IRG1, Lampropoulou et al. confirmed that itaconate acts in an anti-inflammatory manner by inhibiting SDH-mediated oxidation of succinate, impacting on mitochondrial respiration and production of pro-inflammatory cytokines in macrophages *in vitro* and *in vivo* (84). However, another study employing radiolabeling suggests that exogenous DMI does not get taken up by the cell nor processed to itaconate intracellularly. Nonetheless, the presence of DMI in the media still leads to an increase in intracellular levels of itaconate so the authors have postulated that the effects may be due to an unidentified receptor. Therefore, any results obtained using DMI as a source of exogenous itaconate must be interpreted with caution.

### Succinate

Succinate was identified as an oncometabolite before its importance in macrophage metabolism became clear. In 2005, succinate was found to drive the Warburg effect by activating HIF-1α through the inhibition of cytosolic prolyl hydroxylases (85). However, the role of succinate in inflammatory macrophages did not present itself until Tannahill et al. demonstrated that the accumulation of succinate in LPS-stimulated macrophages induced HIF-1α stabilization and activation which in turn leads to an upregulation of pro-inflammatory IL-1β as a target gene (28). This study established that cytosolic HIF-1 prolyl hydroxylases are inhibited upon the accumulation of succinate in normoxic inflammatory macrophages, and confirmed that the IL-1β gene contains HREs in its promoter. The authors also observed a boost in global LPS-induced succinylation, a protein modification akin to malonylation, although the functional significance of the modification is unclear (28).

Another important discovery regarding succinate was the identification of the succinate receptor GPR91, which was previously considered an orphan G-protein coupled receptor (GPCR). A study conducted by He et al. assigned the TCA cycle intermediates succinate and α-ketoglutarate to the orphan GPCRs GPR91 and GPR99, respectively (86). GPR91, which has since been renamed the succinate receptor (SUCNR1), was found to be expressed in a wide variety of tissues (86). SUCNR1 has since been shown to be expressed on macrophages and to play a role in many inflammatory diseases (87–89). Activated M1 macrophages secrete succinate into the extracellular space while also upregulating expression of SUCNR1. This way succinate signals in an autocrine and paracrine manner to stimulate the release of IL-1β. High levels of succinate were found in synovial fluid taken from RA patients and using a *Sucnr1^−/−^* mouse model of arthritis, this succinate feed forward loop was confirmed by impaired macrophage activation as well as reduced levels of IL-1β (87). In contrast, *Sucnr1^−/−^* mice were found to exhibit exacerbated allergic contact dermatitis and in this same study the authors also reported that SUCNR1 deficiency improved arthritis in the mouse model (88). The authors proposed that the increased severity of allergic contact dermatitis observed in the *Sucnr1^−/−^* mice was due to abnormal mast cell development, which leads to mast cell hyperactivation. Recently, the succinate receptor has been implicated in type-2 diabetes and the associated adipose tissue inflammation (89). Succinate levels were raised in both type-2 diabetic patients and mice fed a high-fat diet compared with healthy controls. *Sucnr1^−/−^* mice displayed improved glucose tolerance and had significantly fewer macrophages present in their adipose tissue. In addition, macrophages from the *Sucnr1^−/−^* mice exhibited impaired chemotaxis toward apoptotic adipocytes (89). With more research currently ongoing into the effects of succinate and its receptor in inflammation, the therapeutic potential of targeting SUCNR1 for inflammatory diseases may soon be defined.

While most work has focused on citrate, itaconate, and succinate, other TCA cycle intermediates have also been found to play significant roles in macrophages. Arts et al. demonstrated that an accumulation of fumarate in M1 macrophages, which was dependent on glutaminolysis, was of great importance in trained immunity of macrophages. Fumarate alone induced epigenetic changes akin to those observed in response to β-glucan and also augmented pro-inflammatory cytokine production upon restimulation with LPS (90). This was the first piece of evidence that an accumulation of TCA cycle intermediates can alter the macrophage epigenome but more recently α-ketoglutarate has also been shown to play a part in epigenetic reprogramming in macrophages. It was discovered that α-ketoglutarate, again produced *via* glutaminolysis, is crucial for full M2 activation and drives epigenetic changes in M2-associated genes in a Jumonji Domain Containing Protein 3 (JMJD3)-dependent manner (91). Furthermore, treatment of BMDMs with an inhibitor of glutaminolysis boosted pro-inflammatory cytokine secretion. Ratios of α-ketoglutarate to succinate in an M1 macrophage versus and M2 macrophage differ. There is a larger succinate to α-ketoglutarate ratio in M1 macrophages due to partial blockade of succinate oxidation by SDH. However, in M2 macrophages succinate oxidation proceeds as normal and α-ketoglutarate becomes more important for processes such as epigenetic changes.

As these findings are still recent, more data will no doubt emerge detailing how TCA cycle intermediates and other metabolic changes sculpt the macrophage epigenome.

## Electron Transport Chain (ETC) and Oxidative Phosphorylation

### Overview

**Figure 2:**
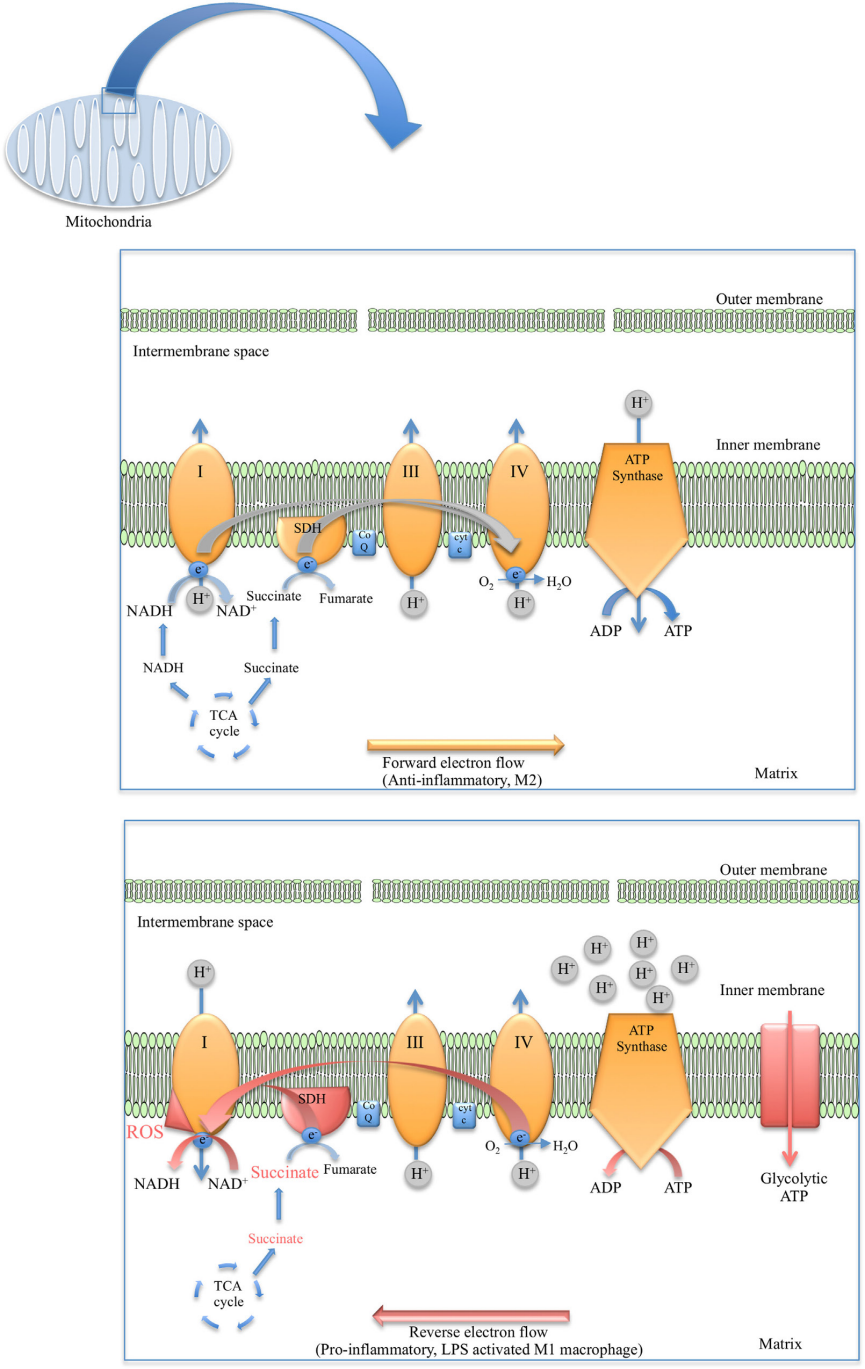
Diagram depicting the flow of electrons in anti-inflammatory macrophages (top) versus the reverse electron transport (RET) phenomenon observed in lipopolysaccharide (LPS)-stimulated macrophages (bottom).

As for the TCA cycle, the reactions of the ETC occur in the mitochondria. The mitochondria have an outer permeable membrane and an inner membrane with extensive folds called cristae. Large electron-carrier complexes in the inner membrane of mitochondria reoxidize NADH and FADH_2_ generated from the TCA cycle, and in the process electrons are passed stepwise to molecular oxygen. During this process protons are taken up from the mitochondrial matrix space and transferred to the intermembrane space. The potential energy of the NADH and FADH_2_ generated during glycolysis and the TCA cycle is thereby used to drive the synthesis of large amounts of ATP as an electrochemical potential gradient for protons is created across the inner mitochondrial membrane (Figure 1). Flow of protons back into the matrix through ATP synthase drives synthesis of ATP. Four large complexes, except cytochrome c and ubiquinone, contain the electron carriers that make up the electron transfer chain. Complex I is the largest of the complexes and contains NADH dehydrogenase responsible for oxidizing NADH. Complex II is composed of four subunits of SDH, the only enzyme that participates in both the TCA cycle and the ETC. SDH is localized on the inner face of the mitochondrial inner membrane where it oxidizes succinate to fumarate through binding of succinate to SDHA, a reaction that is coupled to the reduction of ubiquinone to ubiquinol. As electrons move down the respiratory chain through complex III and complex IV to O_2_, H^+^ ions are transferred to the matrix creating the proton gradient required by the coupled reaction of F_0_F_1_ ATP synthase complex (Figure 2).

### ETC and Macrophage Function

While classically activated macrophages are known to produce most of their ATP *via* glycolysis, alternatively activated macrophages have been shown to utilize OXPHOS. Therefore, M2 macrophages maintain forward electron flow through the ETC and predominantly generate ATP *via* ATP synthase (52). Treatment of macrophages with IL-4 was shown to upregulate OXPHOS *via* the transcription factor STAT6 and PPARγ coactivator-1β (PGC-1β). Overexpression of PGC-1β in BMDMs reduced the production of pro-inflammatory cytokines and PGC-1β knockdown impaired traits of alternative activation such as the promotion of fatty-acid oxidation and arginase activity (92). This effect on OXPHOS is not exclusive to IL-4, as IL-25 was also found to promote M2 polarization and elevate macrophage mitochondrial respiratory capacity (93). IL-10 also stimulates OXPHOS, one way in which the anti-inflammatory cytokine opposes M1 polarization (15).

However, in M1 macrophages OXPHOS is abated and the ETC becomes dysregulated (52). In one study, the downregulation of OXPHOS and the concomitant increase in mitochondrial fragmentation in response to pro-inflammatory stimuli was shown to be under the control of a microRNA–mi-R125b (94). Pro-inflammatory macrophages modify the ETC so that its primary function is ROS production, which is critical in the defense against infection. It is well established that phagosomal ROS is vital for M1 macrophages but the importance of mitochondrial ROS (mROS) has only been appreciated more recently. It was demonstrated that several TLR receptors signal through TRAF6 and evolutionarily conserved signaling intermediate in toll pathway (ECSIT) (which associates with complex I of the ETC) to promote mROS production and the recruitment of the mitochondria to the phagosomes. Perturbation of this signaling pathway was shown to impair bacterial killing by macrophages (95). These findings are not restricted to mice as immune cells isolated from patients suffering from TNF receptor-associated periodic syndrome (TRAPS) were found to have elevated mROS (96). In addition to a more direct role in bacterial killing (95), mROS was also demonstrated to contribute to NLRP3 inflammasome activation in macrophages (97). Further evidence for the importance of mROS in macrophages arises from the role that uncoupling protein 2 (UCP2) appears to play. UCP2 is located at the inner mitochondrial membrane and unlike its homolog UCP1, which is expressed mainly in brown adipose tissue (98), UCP2 is highly expressed in macrophages along with other immune cells. UCP2 is believed to mitigate ROS levels in macrophages through uncoupling of OXPHOS. UCP2*^−/−^* mice were found to be more resistant to *Toxoplasma gondii* infection and macrophages from these knockout mice were found to have elevated of ROS compared with wild-type controls (99). As expected, IL-10 was found to exert the opposite effect on mROS compared with LPS and other TLR agonists. Treatment of macrophages with IL-10 promoted the abolition of dysfunctional mitochondria (characterized by elevated levels of ROS and lower mitochondrial membrane potential) through induction of mitophagy (15). The complexes of the ETC except for complex II (SDH) are known to be able to form supercomplexes in the mitochondrial inner membrane, which seems to change how the electrons are processed depending on the carbon source (100). Supercomplex formation is also thought to restrict mROS production (101). Macrophages were shown to disassemble these supercomplexes in response to bacterial detection, a process which was found to be dependent on TLR signaling and NLRP3 activation (102). Although complex II cannot form supercomplexes, it has been shown that phosphorylation of complex II by Fgr kinase is important in this disassembly (103).

In recent years, it has come to light that mROS in pro-inflammatory macrophages may be generated *via* reverse electron transport (RET) (Figure 2). The first evidence of RET giving rise to mROS was in oxygen sensing (104) and aging in *Drosophila melanogaster* (105). RET at complex I was also demonstrated to drive mROS production in reperfusion injury due to the accumulation of succinate and the elevated activity of SDH (106). Later RET was found to occur in macrophages when Mills et al. established that the build-up of succinate in LPS-stimulated macrophages and the oxidation of this succinate by SDH resulted in the production of mROS, seemingly from RET at complex I (107) (Figure 2). Definite roles have emerged for complex I and complex II in ROS production in M1 macrophages through studies in which inhibitors of these complexes were used. For example, Kelly et al. used metformin and rotenone to inhibit complex I in LPS-treated BMDMs, which markedly reduced mROS production as well as decreased IL-1β and increased IL-10 (108). However, another study used imiquimod and a similar molecule called CL097 to inhibit complex I and NAD(P)H dehydrogenase, quinone 2 (NQO2) and observed a boost in ROS production and NLRP3 activation (109). Inhibiting complex II (SDH) with dimethyl malonate (DMM) inhibits IL-1β production and raises IL-10 levels both in BMDMs and *in vivo*. Expression of alternative oxidase (AOX), which provides a different route for excess electrons so that ROS are not formed, prevents the inflammatory phenotype (107).

M1 macrophages also produce NO, which is induced upon HIF-1α activation (110) and contributes to the bactericidal and antitumor capacity of macrophages (111). NO is known to inhibit mitochondrial respiration *via* complex IV (cytochrome c oxidase) (112, 113), and it may also inhibit complex I through S-nitrosylation of thiol groups on the enzyme (114). This inhibition of mitochondrial OXPHOS has been found to prevent the repolarization of M1 macrophages to an anti-inflammatory M2 phenotype, although the reverse is possible. Inhibition of NO production was shown to significantly improve this repolarization (115). This observation could be clinically relevant as it may be desirable to repolarize M1 macrophages to M2 in the case of inflammatory diseases.

## Fatty-Acid Synthesis and Beta Oxidation

### Overview

The synthesis and degradation of fatty acids occur by two separate processes in different parts of the cell. Fatty-acid synthesis takes place in the cytosol, using citrate from the TCA cycle as a substrate in a series of reactions catalyzed by fatty-acid synthase (FAS). Citrate leaves the mitochondria and the TCA cycle in exchange for malate with the help of the mitochondrial CIC. Cytosolic citrate is then broken down by ACLY into acetyl-CoA and oxaloacetate. While oxaloacetate can be converted back into malate by malate dehydrogenase (MDH) and re-enter the mitochondria, acetyl-CoA on the other hand is converted to malonyl-CoA by acetyl-CoA carboxylase (ACC). Malonyl-CoA can then be polymerized by FAS in a series of repetitive reactions, growing by two carbons with each reaction until it reaches the 16 carbon length of palmitic acid. In addition, acetyl-CoA plays a central role in cholesterol synthesis through the cytosolic mevalonate pathway. Three molecules of acetyl-CoA are condensed to form 3-hydroxy-3-methylglutaryl-coenzyme A (HMG-CoA), which in turn in converted into mevalonate by HMG-CoA reductase, followed by a series of reactions leading to cholesterol.

As beta oxidation or fatty-acid degradation takes place within the mitochondria, the first step involves transport of free cytosolic fatty acids across the mitochondrial membranes. This process starts when fatty acids are converted to acyl-CoA by acyl synthetase, and aided by carnitine palmitoyltransferase I (CPT1) in the outer membrane of the mitochondria, acyl then becomes bound to carnitine. The carnitine–acyl-CPT1 complex enters the mitochondrial matrix aided by acyl carnitine translocase and acyl is finally released with the help of carnitine palmitoyltransferase II (CPT-2), resulting in mitochondrial acyl-CoA. Acyl-CoA is then oxidized in a repetitive cyclic series of reactions with a net yield of each oxidation cycle being one NADH, one FADH_2_, and one molecule of acetyl-CoA.

Malonyl-CoA generated during fatty-acid synthesis serves as a key regulatory feedback loop for beta oxidation as it inhibits the rate-limiting enzyme CPT1, thereby preventing cytosolic fatty acids from binding carnitine and entering the mitochondria where beta oxidation takes place.

### Fatty-Acid Synthesis and Macrophage Function

Overall, fatty-acid synthesis is closely linked to pro-inflammatory effector functions of macrophages. We and others have shown an increase in citrate and fatty acids in LPS-activated macrophages (28, 52). Feingold et al. demonstrated that glucose-derived carbons generated through an increased rate of glycolysis in LPS-activated macrophages were preferentially incorporated into fatty acids and sterols (116). In addition, LPS-activated macrophages, as well as macrophages associated with atherosclerosis, so-called foam cells, display an increased accumulation of triglycerides and cholesterol esters which may contribute to the pathogenesis of chronic inflammatory diseases (117–119). This accumulation is in large due to increased *de novo* synthesis of fatty acids, coupled to a robust increase in several of the key enzymes involved in glycerol lipid synthesis including glutamic-pyruvic transaminase (GPT3), Lipin 1, and diacylglycerol O-acyl transferase 2 (DGAT2). Paired with the marked increase in fatty-acid synthesis observed in LPS-stimulated macrophages is a marked decrease in fatty-acid oxidation, linked with suppressed expression of CPT1 (116). Differentiation of monocytes is linked with an M-CSF-stimulated upregulation of genes required for fatty-acid synthesis, and a switch in major lipid synthesis class from cholesterol in monocytes to phosphatidylcholine in macrophages. This induction of fatty-acid synthesis is critical for monocyte differentiation and phagocytic activity of macrophages. A newly identified protein named FAMIN was found to associate with FAS on peroxisomes and regulate *de novo* lipogenesis. Interestingly, FAMIN was identified through single-nucleotide polymorphisms (SNPs) associated with inflammatory diseases and was found to be essential for the production of pro-inflammatory cytokines and ROS, as well inflammasome activation in LPS-stimulated macrophages and in a murine model of sepsis (120).

### Beta Oxidation and Macrophage Function

In a similar manner to fatty-acid synthesis being coupled to pro-inflammatory macrophages, beta oxidation is synonymous with anti-inflammatory macrophages. Lipolysis liberates free fatty acids, which are taken up by the macrophage by fatty-acid transporters such as CD36, thereby fueling mitochondrial OXPHOS. In IL-4-stimulated macrophages, this metabolic switch is largely mediated through STAT6 and PGC1β (92). Alternatively activated anti-inflammatory M2 macrophages display increased expression of CPT-1, CD36, and medium-chain acyl coenzyme A dehydrogenase (MCAD) (92). M2 polarization depends on lysosomal acid lipase (LAL)-mediated lipolysis as demonstrated by a blocked protective M2 response during parasitic helminth infection (121).

Malonyl-CoA from the TCA regulates fatty-acid oxidation by binding to CPT1, thereby making this the rate-limiting step in beta oxidation. Inflammatory macrophages of adipose tissue contribute to obesity-induced insulin resistance triggered by fatty acids and a range of other stimuli including ROS and pro-inflammatory cytokines. Promoting increased fatty-acid oxidation by over expressing CPT-1 in human adipose tissue macrophages promoted fatty-acid oxidation causing reduced inflammatory responses, as well as improved insulin sensitivity of adipocytes, reduced endoplasmic reticulum stress and less ROS damage in macrophages (122).

Taken together, this indicates that boosting fatty-acid oxidation in inflammatory macrophages would have beneficial anti-inflammatory effects. However, recent studies suggest that the assumption that fatty-acid oxidation is purely anti-inflammatory may be an over simplification. NLRP3 is an important component of one of the large multiprotein inflammasomes. Assembly of the NLRP3 inflammasome occurs in response to a range of different stimuli including viruses, components of bacteria as well as bacterial toxins, liposomes, and cholesterol crystals (123–128). Interestingly, NLRP3 is also activated by palmitate, which through oxidation *via* CPT1, fuels mitochondrial respiration, subsequent production of ROS, and activation of NLRP3 (129–131). Hence, in addition to LPS-activated macrophages requiring fatty-acid synthesis and FAS for adequate activation of NLRP3, oxidation of palmitate is also required for mitochondrial ROS activation of NLRP3. In addition, NLRP3 activation can be inhibited by modulating the activity of NADPH oxidase 4 (NOX4) (129). NOX4 regulates CPT1A activity and fatty-acid oxidation, and inhibition of NOX4 leads to suppressed NLRP3 activity and reduced secretion of IL-1β and IL-18 *in vitro* as well as *in vivo* (132).

Furthermore, as expected, macrophages generated from mice lacking CPT2 displayed impaired fatty-acid oxidation. Surprisingly, however, this did not affect their response to IL-4 polarization (133), implying that fatty-acid oxidation is not required for differentiation of M2 macrophages and that the role for fatty-acid oxidation here is more complex than originally proposed.

## Amino-Acid Metabolism

### Overview

The metabolism of amino acids plays an important role in many cellular processes where free amino acids are used as building blocks for not only protein synthesis but also for *de novo* synthesis of branched chain fatty acids, as in the case of valine and leucine, while glutamine and aspartate are used for purine and pyrimidine synthesis. Furthermore, cellular as well as dietary amino-acid catabolism can be used to support ATP production or as a source of citrate for fatty-acid synthesis. An initial step of transamination, resulting in α-ketoacids, allows for the carbon skeleton of the amino acid to enter the TCA cycle at one of multiple points such as α-ketoglutarate, succinyl-CoA, fumarate, oxaloacetate, pyruvate, or acetyl-CoA, thereby providing fuel in times of superfluous cellular amino acids.

### Amino-Acid Metabolism and Macrophage Function

Cells, including macrophages, of higher vertebrates can synthesize 11 of the 20 amino acids. Dietary intake and protein salvage pathways are the only source of the remaining nine essential amino acids. Three of the essential amino acids, leucine, valine, and isoleucine, are the so-called branched-chain amino acids (BCAAs) with diverse roles outside of nutrition, such as regulation of protein degradation and synthesis in skeletal muscle, as well as regulation of synthesis of neurotransmitters such as serotonin in the brain, thereby affecting behavior. BCAAs also facilitate glucose uptake by the liver and skeletal muscles as well as enhance glycogen synthesis.

The first evidence that amino-acid metabolism can regulate macrophage effector function came with the discovery that macrophages block tumor growth through the consumption of arginine leading to the production of NO (134–138). Since then we have learned that availability and metabolism of several other amino acids such as glutamine and tryptophan also regulate macrophage immune function. The serine/threonine kinase mTOR forms two complexes, mTORC1 and mTORC2, and has been shown to be an important regulator in both innate and adaptive immune cells (139). mTOR plays a key role in macrophages, providing a link between amino-acid availability, coupling this to growth, proliferation, and protein synthesis. Branched-chain ketoacids (BCKAs), a product of BCAA catabolism, has been shown to directly regulate macrophage function by reducing the phagocytic ability of TAMs (140). Further evidence for a role for BCAA comes from data demonstrating that BCAT1, the enzyme responsible for the first step in BCAA catabolism, regulates the metabolic reprogramming in human macrophages. Inhibition of BCAT1 results in decreased glycolysis, oxygen consumption, IRG1 expression, as well as itaconate levels (141). In the interest of space, we will here briefly discuss the role of arginine in macrophage function [amino-acid metabolism in immunity has been reviewed in Ref. (142, 143)].

### Arginine

l-arginine has several key roles in macrophage function. During inflammation, macrophages are responsible for the majority of ROS and nitrogen species produced including NO. This NO production in macrophages, in response to LPS and IFN-γ as well as ILs such as IL-13, requires extracellular l-arginine which enters the cells through specific transmembrane transporters (144–152). Arginine uptake is regulated by pro-inflammatory signals such as IL-1β (149). Once inside the cell, apart from being a precursor in protein synthesis, arginine also acts as a substrate for multiple enzymes including inducible nitric-oxide synthase (iNOS) and arginase, resulting in the production of NO and citrulline, respectively. Both of these metabolic pathways are utilized in macrophages with great opposing effects on immune function, with M1 macrophages utilizing arginine through iNOS resulting in pro-inflammatory NO, and M2 macrophages fluxing arginine *via* arginase resulting in citrulline and a tolerant phenotype associated with wound healing (153). Furthermore, arginase expression in macrophages is linked to limiting inflammatory effector T cell function, as well as correlating with disease severity in visceral leishmaniasis and HIV infection (154, 155). Arginine supplies required for efficient NO output can be restricted by arginase activity, though macrophages can circumvent this by converting L-citrulline to l-arginine, thereby restoring intracellular availability of arginine. However, l-arginine generated in this manner is less effective as a substrate for arginase-derived l-ornithine production of the urea cycle compared with l-arginine originating from the extracellular milieu (156). Arginine metabolism in myeloid cells has been reviewed in depth by Rodriquez et al. (157).

## Concluding Remarks

**Figure 3:**
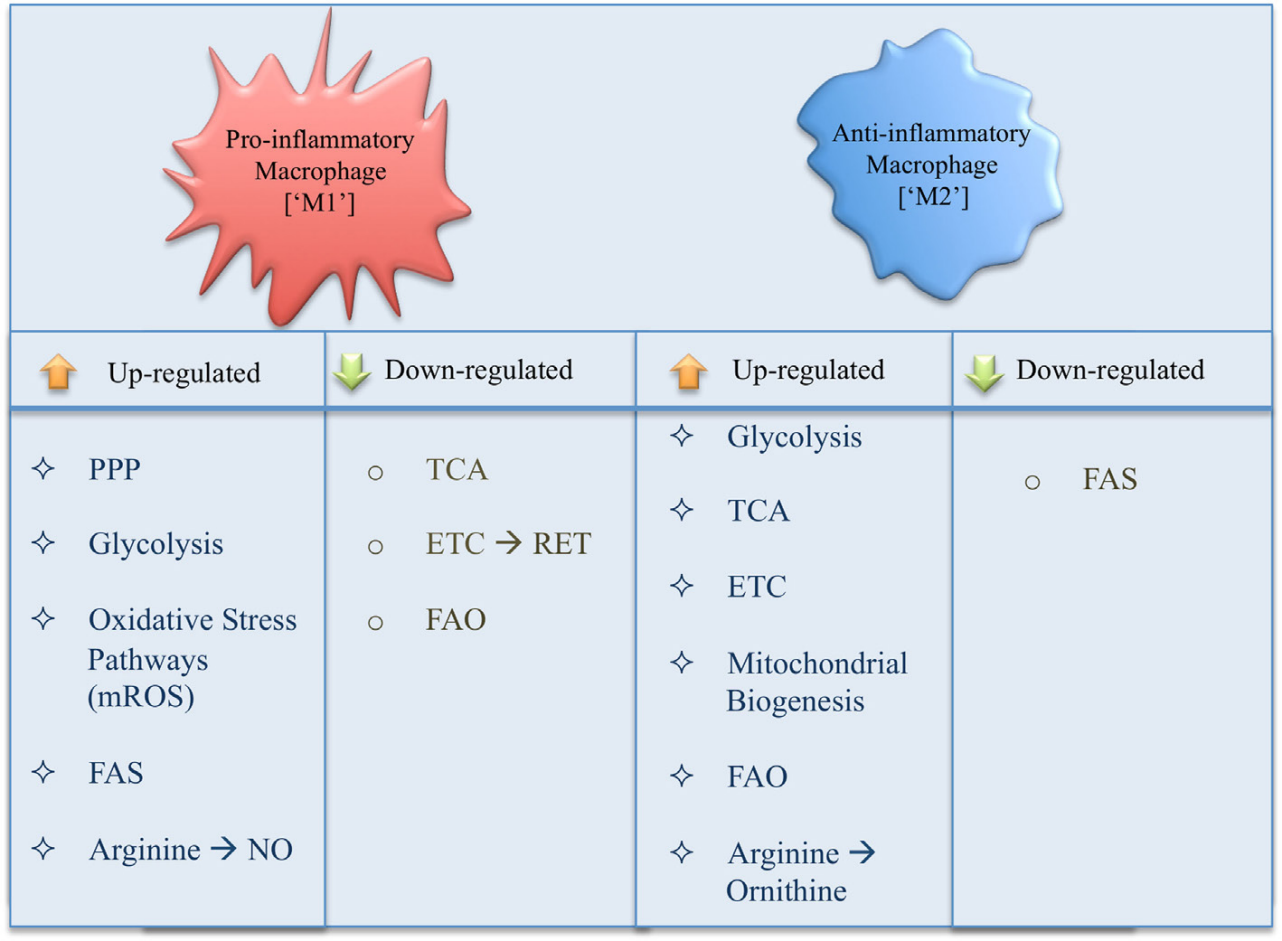
Summary of changes in metabolic pathways occurring in a pro- versus anti-inflammatory macrophage.

The ever-growing literature on immunometabolism demonstrates new roles for metabolic pathways as well as specific pathway intermediates in the metabolic reprogramming of macrophages, leading to profound changes in immune effector functions (Figure 3). In order to simplify and provide an overview of our current understanding of immunometabolism in macrophages, we have here described each pathway as a separate entity; however, in reality these processes are intimately linked. In addition, using a simplified interpretation of macrophage activation gives us a general view of metabolism in inflammatory versus anti-inflammatory macrophages, while reality proves more complex with a spectrum of macrophage subsets occurring in disease. Many questions are still outstanding; however, by its very nature immunometabolism has already firmly established itself as a field, which will provide us with future therapeutic targets for the treatment of immune disorders.

## Author Contributions

All authors listed have made substantial, direct, and intellectual contribution to the work and approved it for publication.

## Conflict of Interest Statement

The authors declare that the research was conducted in the absence of any commercial or financial relationships that could be construed as a potential conflict of interest.

## Abbreviations

ACC, acetyl-CoA carboxylase; ACLY, ATP citrate lyase; ACO2, aconitase 2; AOX, alternative oxidase; BCAA, branched-chain amino acid; BCKA, branched-chain keto acid; BMDM, bone-marrow-derived macrophage; CAD, *cis*-aconitate decarboxylase; CARKL, carbohydrate kinase-like protein; CIC, citrate carrier; CPT1, carnitine palmitoyltransferase 1; DGAT2, diacylglycerol O-acyl transferase 2; DMI, dimethyl itaconate; DMM, dimethyl malonate; ECAR, extracellular acidification rate; ECSIT, evolutionarily conserved signaling intermediate in toll pathway; EIF2AK2, eukaryotic translation initiation factor-2 alpha kinase 2; ETC, electron transport chain; FAS, fatty-acid synthase; FIH, factor-inhibiting HIF; GAIT, gamma-interferon-activated inhibitor of translation; GAPDH, glyceraldehyde-3-phosphate dehydrogenase; GLUT1, glucose transporter 1; GM-CSF, granulocyte-macrophage colony-stimulating factor; GPCR, G-protein-coupled receptor; GPT3, glutamic-pyruvic transaminase; HIF, hypoxia-inducible factor; HMG CoA, 3-hydroxy-3methylglutaryl-coenzymeA; HO-1, heme oxygenase 1; HRE, hypoxia response element; IDH, isocitrate dehydrogenase; IFN-γ, interferon gamma; IL, interleukin; iNOS, inducible nitric-oxide synthase; LAL, lysosomal acid lipase; LDH, lactate dehydrogenase; LIF, leukemia inhibitory factor; LPS, lipopolysaccharide; MCAD, medium-chain acyl coenzyme A dehydrogenase; M-CSF, macrophage colony-stimulating factor; MDH, malate dehydrogenase; mTOR, mechanistic target of rapamycin; NLRP3, nod-like receptor family pyrin domain containing 3; NO, nitric oxide; NOX4, NADPH oxidase 4; OCAR, oxygen consumption rate; OXPHOS, oxidative phosphorylation; PBMC, peripheral blood mononuclear cells; PDH, pyruvate dehydrogenase; PDK1, pyruvate dehydrogenase kinase 1; PD-L1, programmed death-ligand 1; PGC-1β, PPAR-gamma coactivator 1β; PGE2, prostaglandin E2; PKM2, pyruvate kinase M2; PPP, pentose phosphate pathway; PRR, pattern recognition receptor; RA, rheumatoid arthritis; RET, reverse electron transfer; ROS, reactive oxygen species; SDH, succinate dehydrogenase; STAT, signal transducer and activator of transcription; SUCNR1, succinate receptor 1; TAM, tumor-associated macrophage; TCA, tricarboxylic acid; TLR, toll-like receptor; TNF-α, tumor necrosis factor-alpha; UCP, uncoupling protein; VEGF, vascular endothelial growth factor; VHL, von Hippel–Lindau protein; 2-DG, 2-deoxyglucose; 3′UTR, 3′untranslated region.
